# ESpma: A method for assessing biological/non‐biological interfaces using point‐clouds‐based structural features and protein language models

**DOI:** 10.1002/pro.70790

**Published:** 2026-09-17

**Authors:** Sarah Nozawa, Kentaro Tomii, Yoshinori Fukasawa

**Affiliations:** ^1^ Center for Bioscience Research and Education Utsunomiya University Utsunomiya Japan; ^2^ Artificial Intelligence Research Center National Institute of Advanced Industrial Science and Technology (AIST) Tokyo Japan

**Keywords:** deep learning, interfaces, point cloud, protein complexes, protein language model

## Abstract

Distinguishing biological protein–protein interfaces from non‐biological contacts remains an important task in structural biology, particularly as protein complex structures continue to accumulate. Recent advances in protein language models (pLMs) have expanded their use across diverse protein prediction tasks, including sequence‐based protein–protein interaction prediction. Such approaches often pool representations over entire sequences, whereas structure‐based methods commonly rely on more complex graph‐ or geometry‐based integration. We therefore asked how much interface‐relevant information could be extracted by simply pooling pLM embeddings over structurally defined local regions. Here, we revisited biological‐versus‐crystal interface classification as a structurally well‐defined testbed for this question. We developed a framework that compares full‐sequence pooling, interface‐localized pooling of pLM embeddings, and multimodal integration of sequence‐derived embeddings with point‐cloud representations of protein surfaces. On two benchmark datasets, interface‐localized pooling achieved stronger performance than full‐sequence or non‐interacting surface pooling across three pLM backbones. Despite its simplicity, the resulting representation performed within the range of established methods that rely on explicit evolutionary analysis or geometric modeling. Incorporating explicit geometric surface information changed performance slightly, without reaching statistical significance. Because the model is linear, the interface‐level score decomposes exactly into per‐residue contributions, which varied within amino‐acid types. Together, our results indicate that the principal gain arises from localizing pLM embeddings to physically interacting residues, enabling a lightweight and accessible implementation for biological‐versus‐crystal interface classification. Code and scripts are available on GitHub: https://github.com/fukasawa-group/espma.

## 1 INTRODUCTION

Protein crystal structures are an important resource for understanding protein function because they can reveal the structural basis of functional assemblies, commonly termed biological assemblies or biological units. However, the crystal lattice also contains non‐biological interfaces generated by crystal packing. Distinguishing biological interfaces from crystal contacts is therefore a fundamental problem in the interpretation of oligomeric states derived from X‐ray crystallography (Capitani et al., 2016; Krissinel & Henrick, 2007). Large crystal contacts resemble biological interfaces in their physicochemical properties, which makes classification harder (Janin, 1997; Luo et al., 2015).

This challenge has traditionally been addressed using physicochemical, structural, and evolutionary features (Bernauer et al., 2008; Elcock & McCammon, 2001; Jiménez‐García et al., 2019; Krissinel & Henrick, 2007; Valdar & Thornton, 2001). More recently, deep‐learning approaches have expanded the range of available representations. In particular, protein language models (pLMs) have substantially improved sequence‐derived representations and achieved strong performance across a wide range of protein prediction tasks (Brandes et al., 2022; Elnaggar et al., 2023; Heinzinger et al., 2024; Lin et al., 2023), including interaction‐related classification (Bernett et al., 2024; Reim et al., 2025; Yang et al., 2024). At the same time, the success of AlphaFold demonstrated that rich structural information can be inferred from sequence‐derived evolutionary signals, further motivating the use of learned protein representations in problems related to molecular recognition and assembly (Gazizov et al., 2026; Jumper et al., 2021). Recent studies have reported that modern pLMs do not necessarily benefit from the addition of external evolutionary information, suggesting that modern pLMs may already encode rich evolution‐related information in their internal representations (Erckert & Rost, 2024). Similarly, protein embeddings learned in the AlphaFold2 model are transferable into the ligand‐binding‐site prediction with a simple operation (Gazizov et al., 2026). Whether further gains require richer geometric input or better extraction from the embeddings remains an open question.

Many pLM‐based approaches represent proteins primarily at the whole‐sequence level (Liu et al., 2025) owing to their simplicity. By contrast, recent studies have increasingly combined pLM‐derived features with structure‐based representations such as interface graphs (Hu & Ohue, 2025; Wu et al., 2023; Xu & Bonvin, 2024) and point‐cloud‐based geometric learning frameworks (Arteaga & Poptsova, 2025). These multimodal approaches aim to complement sequence‐derived information with explicit structural context. However, their relative importance for interface‐related prediction tasks has not been clearly discussed. In this context, structural information can contribute at two distinct levels. First, it can serve to define the locality of the prediction target, identifying which residues participate in the interface. Second, it can provide explicit geometric features that directly encode the shape and physicochemical properties of the interface surface. Whether these two levels of structural contribution are equally important, or whether one dominates, has not been clearly disentangled in existing approaches.

To address this question, we revisited biological‐versus‐crystal interface classification using a deep‐learning framework designed to disentangle the effects of interfacial locality and structural information. Specifically, we formulated the task as a binary classification problem in which each interface between a pair of protein chains was assigned as either biological or crystallographic. We considered this problem a suitable testbed because both positive and negative examples correspond to physically realizable interfaces, allowing the contribution of structural information to be assessed in a comparatively well‐defined setting. To this end, we compared interface‐localized pooling of residue‐level pLM embeddings with full‐sequence pooling and multimodal models incorporating point cloud‐based structural representations. Based on these comparative analyses, we developed and publicly released a practical tool for biological‐versus‐crystal interface classification.

## 2 RESULTS

### 2.1 Interface‐localized pooling improves biological‐interface classification

**FIGURE 1:**
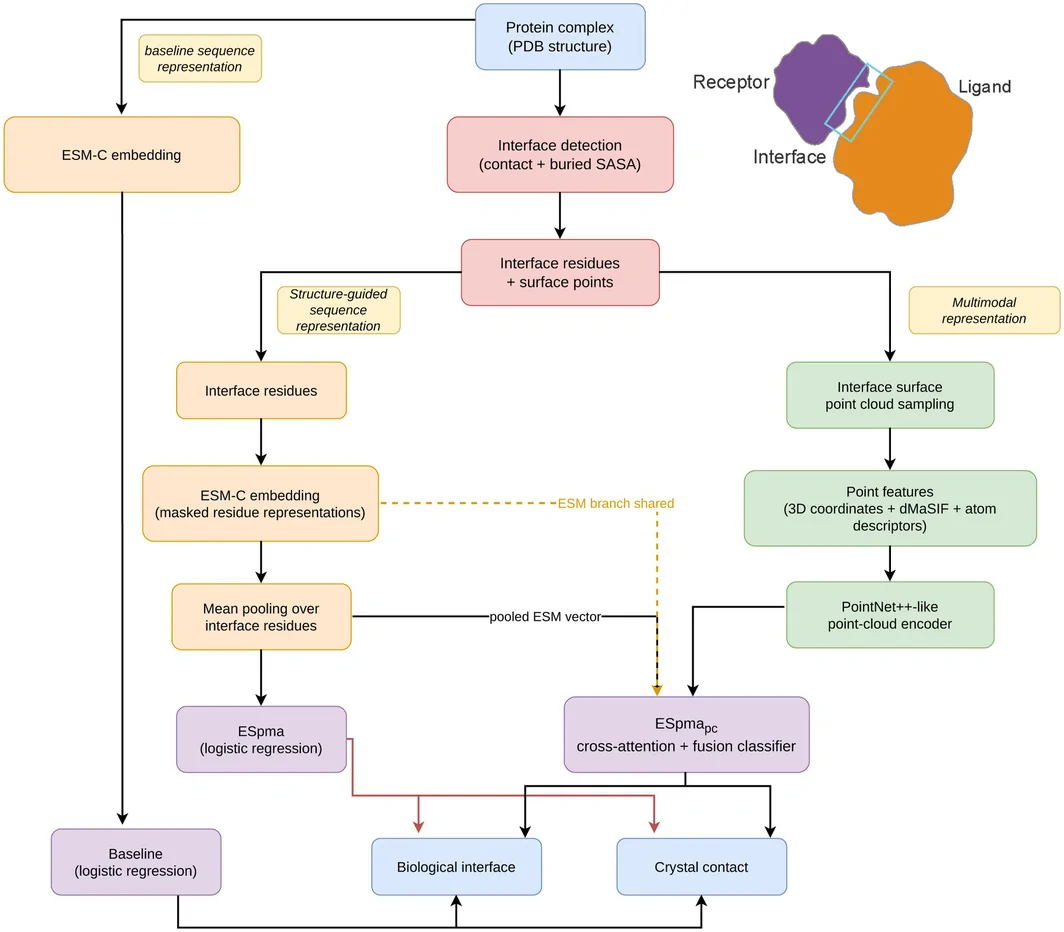
Overview of the interface classification workflow. Two complementary modeling approaches were evaluated. The sequence‐based baseline model used pooled ESM‐C embeddings from the full‐length sequences, whereas the ESpma model used residue embeddings pooled over interface residues only. In the multimodal model (ESpma_pc_), structural representations were derived from surface point clouds sampled from the interface region and combined with ESM embeddings.

To systematically evaluate the contribution of sequence‐ and structure‐derived information for distinguishing biological interfaces from crystal contacts, we developed a workflow that combines interface extraction from experimental structures with machine learning models operating on interface‐conditioned representations (Figure 1).

Starting from protein complexes obtained from crystallographic structures, candidate interfaces were first identified based on intermolecular contacts and buried solvent‐accessible surface area. Specifically, residue pairs within 8 Å across chains were considered potential interface contacts, and residues contributing at least 1 Å^2^ (Capitani et al., 2016) of buried surface area (BSA) were defined as interface residues. These residues define the interacting region used to construct both sequence‐based and structure‐based representations. To determine whether the predictive benefit arose specifically from localizing pLM representations to the interacting interface, rather than merely from restricting pooling to fewer or surface‐exposed residues, we compared interface‐localized pooling with full‐sequence pooling and size‐matched non‐interacting surface pooling.

Interface residues were encoded using the ESM‐C model, and residue‐level embeddings were aggregated by mean pooling over interface residues to obtain a fixed‐length representation of the interface. This representation was used to train a simple logistic regression classifier, denoted ESpma (ESM‐based prediction model for assessing the reliability of protein–protein interfaces). We first examined how the pooling region affected the predictive utility of residue‐level pLM embeddings. Using the same pLM embeddings and logistic‐regression classifier, we compared ESpma with pooling over the full sequences (baseline1) and non‐interacting surface residues (baseline2). Interface‐localized pooling (ESpma) achieved the highest AUROC and classification performance, outperforming both whole‐sequence and non‐interacting surface pooling (Table 1). The same overall trend was observed with other comparably sized pLMs, including ESM‐2 650M and Ankh‐base, indicating that the benefit of interface‐localized pooling was not specific to ESM‐C (Table 1). Thus, the improvement could not be explained solely by restricting the representation to surface residues and instead appeared to reflect information specifically enriched at the interaction interface.

**TABLE 1:** Performance of pooling strategies on the redundancy‐controlled benchmark.

Method	Sen	Spe	ACC	MCC	AUROC	*p*‐value
Baseline1^a^	0.556	**0.938**	0.747	0.535	0.832	0.007
Baseline2^a^	0.605	0.901	0.753	0.530	0.819	0.002
ESpma^a^	**0.778**	0.914	**0.846**	**0.698**	**0.912**	—
Baseline1^b^	0.556	**0.938**	0.747	0.535	0.829	0.011
Baseline2^b^	0.605	0.901	0.753	0.530	0.842	0.044
ESpma^b^	**0.778**	0.926	**0.852**	**0.712**	**0.899**	—
Baseline1^c^	0.444	**0.938**	0.691	0.440	0.812	0.011
Baseline2^c^	0.556	0.864	0.710	0.441	0.794	0.007
ESpma^c^	**0.765**	0.901	**0.833**	**0.673**	**0.892**	—

*Note:* Classification performance of the baseline models and the proposed models across three pLM backbones. Sen, sensitivity; Spe, Specificity; ACC, accuracy; MCC, Matthews correlation coefficient; AUROC, area under the receiver operating characteristic curve; Biological interfaces were treated as the positive class. Baseline1: Mean pooling over the full‐length sequences of the two interacting chains. Baseline2: Mean pooling over randomly sampled non‐interface surface residues, matched to the number of interface residues. ESpma: Mean pooling over structurally defined interface residues. *p*‐values were calculated using DeLong's test by comparing each AUROC with that of the corresponding ESpma model. ESM‐2 and ESM‐C control models coincide in threshold‐dependent metrics but differ in their predictions, disagreeing on 12 (Baseline1) and 18 (Baseline2) of 162 complexes. AUROC values differ accordingly. Bold values indicate the highest value for each metric within each pLM backbone.

^a^ ESM‐C 600M.

^b^ ESM‐2 650M.

^c^ Ankh‐base.

### 2.2 Robustness to alternative interface definitions

To assess the robustness of interface locality, we evaluated alternative interface definitions using different heavy‐atom distance and buried surface area (BSA) thresholds (Table S2). Models were evaluated using combinations of 5‐ or 8‐Å distance cutoffs and BSA thresholds of 0, 1, or 10 Å^2^ (Capitani et al., 2016). Performance was generally stable across these definitions (Table S2). Although the 8‐Å condition without a BSA filter showed a lower point estimate, the overall results indicate that the pooling benefit was not specific to a narrowly selected cutoff.

### 2.3 Explicit geometry provides modest and dataset‐dependent incremental benefit

We next assessed whether explicit geometric information provided additional predictive value beyond interface‐localized pLM pooling. For this purpose, we evaluated a surface‐based point cloud model (PC) designed to encode the local geometry and physicochemical context of the molecular interface using the PointNet++ framework (Qi et al., 2017) (Figure S3). The point‐cloud representation was then integrated with interface‐localized pLM embeddings to form the multimodal ESpma_pc_ model. Because the two models differ in input type and classifier, ESpma_pc_ is used here as a geometry‐aware comparator rather than as an architecture‐matched ablation of ESpma. On the primary benchmark, ESpma_pc_ performed comparably to ESpma in AUROC, and the difference was not statistically significant (Table 2). The incremental benefit of adding explicit geometry was therefore substantially smaller than the gain obtained by restricting pLM pooling to the interacting interface (Table 1).

**TABLE 2:** Contribution of explicit geometric features beyond interface‐localized pLM representations.

Method	Sen	Spe	ACC	MCC	AUROC	*p*‐value
ESpma	0.778	**0.914**	**0.846**	**0.698**	**0.912**	—
PC	**0.889**	0.642	0.765	0.548	0.885	0.266
ESpma_pc_	0.840	0.840	0.840	0.679	0.906	0.580

*Note:* Performance comparison of ESpma, which uses structural information to localize the pooling of pLM embeddings; PC, which models interface geometry using point‐cloud features alone; and ESpma_pc_, which augments ESpma with explicit point‐cloud‐based geometric features. Abbreviations are as defined in Table 1. *p*‐values were calculated using DeLong's test by comparing AUROC with that of the ESpma model. Bold and underlined values indicate the highest and second‐highest values, respectively, for each metric.

### 2.4 Comparison with established interface‐classification methods

The redundancy‐controlled ESpma and ESpma_pc_ models were benchmarked against established interface‐classification methods on the primary dataset (Table 3). As previously demonstrated, ESpma substantially increased performance relative to the baselines. ESpma also achieved higher performance in metrics than several dedicated methods. Pairwise comparisons nominally favored ESpma over PRODIGY‐crystal and DeepRank‐GNN in AUROC (Table 3). Thus, ESpma reached the upper performance range of substantially more complex interface‐classification methods using only interface‐localized mean pooling and regularized logistic regression.

**TABLE 3:** Performance comparison of classification methods.

Method	Sen	Spe	ACC	MCC	AUROC	*p*‐value
ESpma	0.778	**0.914**	**0.846**	**0.698**	**0.912**	—
ESpma_pc_	0.840	0.840	0.840	0.679	0.906	0.580
PRODIGY‐crystal	**0.926**	0.543	0.735	0.508	0.843	0.027
EPPIC	0.914	0.741	0.827	0.664	0.899	0.686
DeepRank‐GNN	0.813	0.827	0.820	0.640	0.865	0.031
DeepRank‐GNN‐esm	0.765	0.901	0.833	0.673	0.889	0.254

*Note:* Classification performance of the proposed models and previously reported methods for distinguishing biological interfaces from crystal contacts. Abbreviations are as defined in Table 1. *p*‐values were calculated using DeLong's test by comparing AUROC with that of the ESpma model. Bold and underlined values are defined as in Table 2.

Comparable classification performance was also observed relative to methods that provide only discrete predictions, including PISA, although the absence of continuous scores precluded ROC‐based pairwise comparisons (Table S3). Among contemporary methods for biological‐versus‐crystal interface classification, EPPIC and DeepRank‐GNN‐esm represented particularly relevant benchmarks. DeepRank‐GNN‐esm provided the most direct methodological comparison because it integrates pLM embeddings with graph neural‐network representations and additional interface features.

### 2.5 Evaluation on the Zhu dataset

Because EPPIC provided a concordant and established performance on the primary benchmark and DeepRank‐GNN‐esm represented the most direct pLM‐based comparator, we further evaluated these methods on the additional redundancy‐controlled Zhu dataset.

**FIGURE 2:**
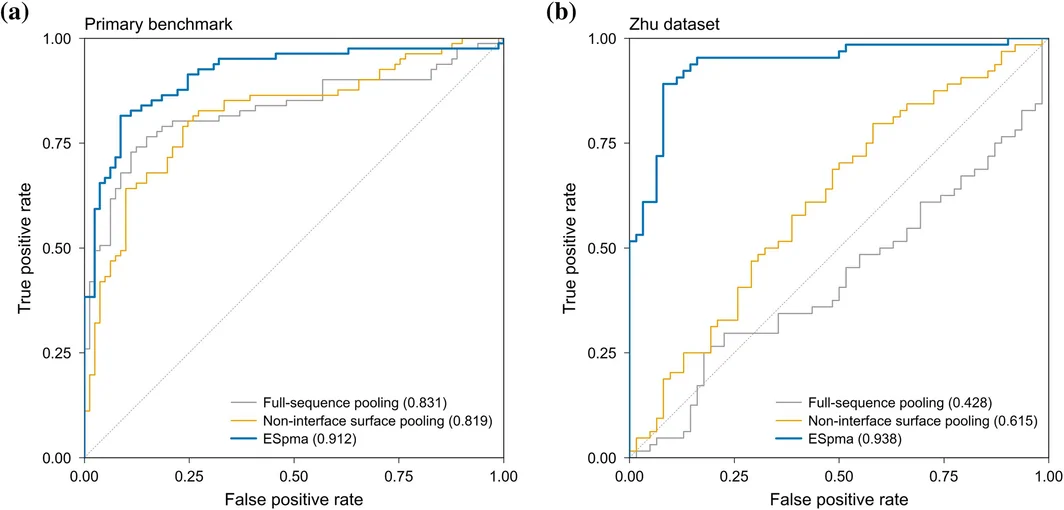
ROC curves for interface classification under different pooling strategies. Performance using the ESM‐C backbone on (a) the primary benchmark and (b) the Zhu dataset, comparing full‐sequence pooling, non‐interface surface pooling, and interface‐localized pooling (ESpma). AUROC values are given in parentheses. The diagonal indicates chance performance. In the Zhu dataset, the two classes largely share the same chain sequences, whereas in the primary benchmark, they are drawn from different structures. Corresponding results for ESM‐2 and Ankh‐base are given in Tables 1 and S4.

On this dataset, interface‐localized pooling consistently outperformed both whole‐sequence and size‐matched non‐interface surface pooling across ESM‐C 600M, ESM‐2 650M, and Ankh‐base (Figure 2; Tables 4 and S4). The reproducibility of this trend across multiple pLMs and two benchmarks suggests that the benefit of interface‐localized pooling was not specific to either ESM‐C or the primary test dataset.

**TABLE 4:** Performance comparison on the Zhu benchmark.

Method	Sen	Spe	ACC	MCC	AUROC	*p*‐value
Baseline1	0.359	0.565	0.460	−0.078	0.428	<0.001
Baseline2	0.516	0.613	0.563	0.129	0.615	<0.001
ESpma	0.672	0.935	0.802	0.628	0.938	—
ESpma_pc_	0.781	0.952	0.865	0.742	**0.957**	0.145
EPPIC	**0.875**	0.968	**0.921**	**0.845**	0.955	0.419
DeepRank‐GNN‐esm	0.609	**1.000**	0.802	0.659	0.802	<0.001

*Note:* Performance of ESpma and selected interface‐classification methods on the redundancy‐controlled Zhu dataset. Abbreviations are as defined in Table 1. *p*‐values were calculated using DeLong's test by comparing AUROC with that of the ESpma model. Bold and underlined values are defined as in Table 2.

Among the ESM‐C‐based models, ESpma achieved a significantly higher AUROC than that of DeepRank‐GNN‐esm (Table 4). ESpma_pc_ showed a further numerical increase over ESpma, although the difference between the two proposed models was not statistically significant here as well. EPPIC achieved the highest threshold‐based classification performance, particularly in sensitivity, whereas its AUROC was comparable to those of ESpma and ESpma_pc_ and did not differ significantly from that of ESpma (Table 4).

Taken together, ESpma reached the performance range of substantially more complex methods on both benchmarks, using only interface‐localized mean pooling and regularized logistic regression. On the Zhu dataset, its AUROC did not differ significantly from that of EPPIC, which relies on homolog searches and evolutionary analysis, or from that of the multimodal ESpma_pc_. These results indicate that simple interface‐localized pooling can reach the performance of methods relying on explicit geometry or on explicit evolutionary analysis.

### 2.6 Residue‐level characterization of the interface‐classification signal

**FIGURE 3:**
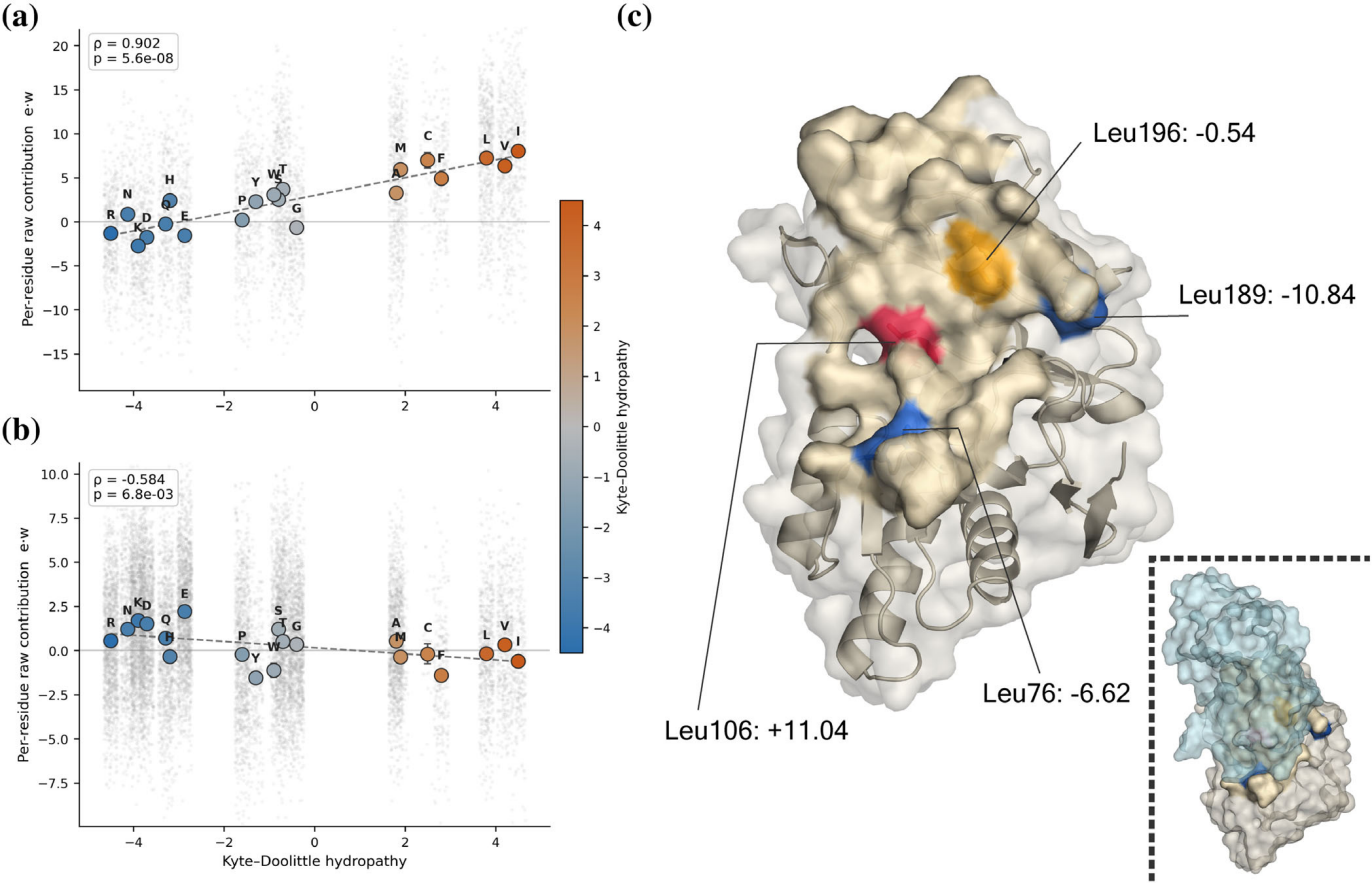
Per‐residue contribution to interface classification as a function of amino‐acid hydrophobicity. Per‐residue contributions cᵢ (Methods) are shown for residues from the 81 biological interfaces of the test set; positive values favor the biological class. (a) Interface residues, scored with ESpma. (b) All non‐interacting surface residues, scored with the baseline2 model. In (a) and (b), markers are per‐residue‐type means colored by Kyte–Doolittle hydropathy (error bars, s.e.m.); gray points are individual residues. Dashed line, OLS fit; ρ, Spearman correlation over the 20 means of amino acids. (c) Score dependence on position within the interface. The four interface leucines of one 1UJ6 (Hamada et al., 2003) chain are colored by cᵢ, ranging from Leu106 near the interface center (+11.34) to Leu189 at the periphery (−10.84).

To examine whether the interface‐classification signal could be represented by a sparse set of potentially interpretable features, we evaluated a pretrained SAE for ESM‐C. However, SAE‐based models retained only part of the predictive signal (Table S5). We therefore analyzed the original embeddings directly by exploiting the linearity of mean pooling and logistic regression, which allowed the classifier score to be decomposed exactly into residue‐level effects. Their amino‐acid‐level averages correlated with hydrophobicity (Figure 3a,b), consistent with the compositional differences previously reported between interfaces and non‐interface surfaces (Bahadur et al., 2004). However, contributions varied substantially even within the same amino‐acid type (gray points in Figure 3a), indicating that the signal is not determined by amino‐acid identity alone.

As an illustrative example, one chain of the 1UJ6 homodimer contained four leucine residues at the interaction interface that spanned a wide range of residue‐level scores: Leu106 (+11.04), Leu196 (−0.54), Leu76 (−6.62), and Leu189 (−10.84). Despite sharing the same amino‐acid identity, their scores showed a clear spatial pattern. Leu106 was located near the interface center, Leu76 and Leu189 near the periphery, and Leu196 at an intermediate position (Figure 3c). This example illustrates that residue‐level effects were spatially organized within an experimentally observed interface and were not determined by amino‐acid identity alone.

### 2.7 Docking decoys as case studies of locality preservation

To investigate whether model performance depends on how well interface locality is defined, we next examined docking decoy sets from the Dockground benchmark (Liu et al., 2008). Because ESpma pools embeddings only over pose‐defined interacting residues, poses recovering more native contacts were expected to include more residues characteristic of the native interface and consequently receive higher scores.

Following a previous study (Collins et al., 2024), we categorized cases according to the extent of conformational change between the unbound and bound states. We therefore selected 1R0R, 1GPW, and 1F6M from the Dockground decoy set as representative easy, intermediate, and difficult examples, respectively. The bound–unbound Cα RMSDs of the receptor and ligand were 1SCN (0.75 Å) and 2GKR (1.14 Å) for 1R0R, 1THF (3.56 Å) and 1K9V (0.69 Å) for 1GPW, and 1TRB (7.29 Å) and 2TRX (1.00 Å) for 1F6M, consistent with progressively larger structural deviations across these case studies. In these case studies, the relationship between predicted scores and the fraction of native contacts (fnat) depended on the difficulty of the docking problem.

**FIGURE 4:**
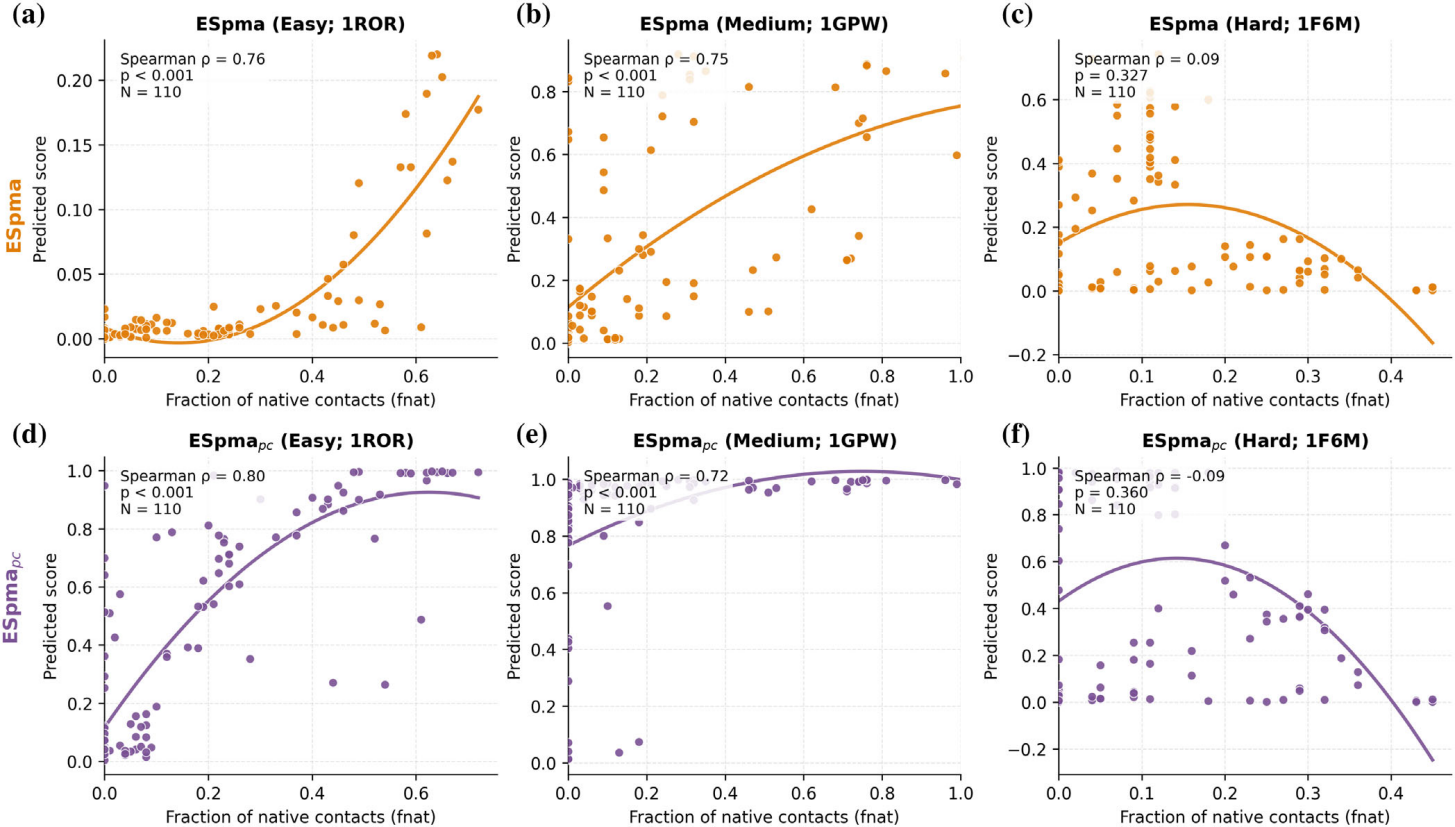
Performance of ESM‐based and multimodal models on docking decoy sets of varying difficulty. Each panel shows the relationship between the fraction of native contacts (fnat) and predicted model scores for individual docking decoys. Panels (a)–(c) correspond to ESpma and panels (d)–(f) to ESpma_pc_. In the easy and intermediate cases (a–b and d–e), both models showed positive correspondence with fnat, consistent with effective scoring when decoy interfaces remained reasonably consistent with the native bound form. In the difficult case, neither model showed meaningful correlation with fnat, reflecting the loss of a native‐like local interaction context.

For the relatively easy case (1R0R) in which unbound structures closely resemble the bound conformation, scores derived from ESpma embeddings and ESpma_pc_ showed positive correlations with fnat (Figure 4a,d). This suggests that sequence‐derived signals captured by the language model can identify native‐like interfaces when docking poses remain close to the native state. In the intermediate case (1GPW), both models also showed positive correlations with fnat, suggesting that these representations remained effective when interface locality was still reasonably preserved (Figure 4b,e).

However, in difficult cases containing highly non‐native interfaces (1F6M), neither model showed meaningful correlation with fnat, underscoring that the locality used to extract information from the internal representations becomes unreliable in these regimes (Figure 4c,f). We should note that predicted probabilities were often saturated near 0 or 1 in these docking experiments and should therefore be interpreted primarily as relative ranking scores rather than calibrated probabilities. These examples therefore illustrate the sensitivity of the representation to interface definition rather than the performance of ESpma as a scoring function.

## 3 DISCUSSION

Distinguishing biologically relevant protein interfaces from crystal packing contacts has been studied extensively using structural, energetic, and evolutionary features (Bahadur et al., 2004; Da Silva et al., 2015; Fukasawa & Tomii, 2019; Krissinel & Henrick, 2007; Liu et al., 2014; Ponstingl et al., 2003; Xu & Dunbrack Jr., 2011). In this study, we revisited this classical problem as a demonstrative case for modern protein representations.

A key finding of this study is that structural information contributes to interface classification primarily by defining appropriate locality. Using structural information to identify interface residues and restrict the pooling scope of pLM embeddings (ESpma) yielded the largest improvement over the sequence‐only baseline and the size‐matched non‐interface surface control (Table 1). In contrast, adding explicit geometric surface features (ESpma_pc_) changed AUROC only slightly, and the difference did not reach statistical significance on either benchmark (Tables 2 and 4). In ESpma, structure is used only to select which residues are pooled, and this requires only contact‐level geometry. The pooled representation itself contains no coordinates or interatomic distances. In contrast, ESpma_pc_ adds an explicit description of surface shape and physicochemical context. The comparison with PC and ESpma_pc_ therefore isolates the contribution of explicit geometric description from that of the selection itself. Explicit geometric description without interface definition cannot be constructed, because the definition of interface locality is a prerequisite for a point‐cloud representation.

The importance of locality was more apparent when models were transferred to another dataset. On the Zhu dataset, full‐sequence pooling and non‐interface surface pooling performed at or only slightly above chance level (Tables 4 and S4). Crystal contacts were defined in the same biological complexes for this dataset, so the two classes largely share the same chain sequences. However, ESpma retained its performance (0.92 to 0.94).

Pooling over the entire sequence dilutes interface‐relevant signals. The size‐matched non‐interface surface control performed worse than ESpma, so the benefit cannot be attributed to restricting the representation to fewer residues. Classification of crystal contacts may be a favorable setting for locality. Because biological interfaces and crystal contacts differ, the relevant residues are concentrated at particular and definable surface regions (Table S2). Consistent with this, pooling strategies that preserve local context have been reported to matter in other pLM applications (Hoang & Singh, 2025).

Classification based on high‐dimensional signals presents challenges regarding interpretability. To address this, we analyzed performance using low‐dimensional features using SAE; however, this task shows a non‐negligible degree of signal loss (Table S5). In this context, ESpma offers an alternative approach to interpretation due to its linearity. Because mean pooling and logistic regression are both linear, the classifier score decomposes exactly into per‐residue contributions. Their amino‐acid‐level averages correlated with hydrophobicity (Figure 3a), consistent with the compositional differences reported previously (Bahadur et al., 2004). However, contributions varied substantially within each amino‐acid type (Figure 3a,b). This indicates that the signal is not determined by amino‐acid identity alone. As an illustration, the four interface leucines of one 1UJ6 chain received markedly different contributions depending on their location within the interface (Figure 3c).

The same dependence is visible when the interface itself is varied rather than the sequence. Because the two chain sequences are identical across all poses of a docking decoy set, any variation in score arises solely from the set of residues selected for pooling. Scores correlated with fnat only for cases in which the local interface arrangement remained close to the native bound form (Figure 4). A systematic assessment of ESpma as a scoring function would require large‐scale evaluation on benchmarks such as CAPRI. We have therefore limited our claims to the illustrative role of these examples.

A practical advantage of ESpma is its simplicity. Methods incorporating evolutionary information, such as EPPIC, remain powerful for this task. However, they typically require sequence‐database searches, whose computational cost may increase as reference databases continue to grow. Embedding extraction with a mid‐sized pLM and subsequent mean pooling required on the order of seconds per interface, and prediction using logistic regression was effectively instantaneous. To facilitate practical use, we provide the trained classifiers together with a Google Colab environment that allows users to run the method without installing specialized dependencies. This lightweight design lowers the barrier for applying modern protein language model–based approaches to structural interface analysis.

Overall, this study highlights that a classical structural biology problem can be effectively revisited using modern protein representations. Although explicit geometric modeling remains informative (Table 2), our findings indicate that it did not add significantly to interface‐localized embeddings. The findings further suggest that model performance depends not only on the representation itself but also on how spatial locality is defined and incorporated. Future work should explore more integrated combinations of sequence‐derived and geometric representations, particularly for challenging regimes such as docking evaluation or the assessment of predicted complex structures.

## 4 MATERIALS AND METHODS

### 4.1 Problem formulation

Protein–protein interfaces observed in crystal structures arise either from biologically relevant assemblies or from crystal packing artifacts. Distinguishing these two classes is essential for correctly interpreting oligomeric states derived from X‐ray crystallography. In this work, the task is formulated as a binary classification problem in which each interface between two protein chains is classified as either a biological interface or a crystal contact. Interfaces were defined at the level of chain pairs extracted either from the asymmetric unit (ASU) or from crystallographic symmetry mates. Each interface therefore represents a pair of chains interacting within the crystal lattice. The classification task is performed at the interface level rather than at the residue level.

### 4.2 Dataset preparation

Training data were derived from the dataset compiled by Baskaran et al. (2014). This dataset contains interfaces extracted from Protein Data Bank structures and annotated as biological assemblies or crystal packing contacts based on evolutionary conservation analysis.

The dataset used in this study contains 2829 biological interfaces and 2921 crystal interfaces, providing a nearly balanced classification problem. Interfaces were identified using geometric and solvent accessibility criteria. Two chains were considered to form an interface if at least one pair of residues across the chains had heavy‐atom distance of 8 Å or less and if the buried solvent accessible surface area exceeded 1 Å^2^ (Capitani et al., 2016). Solvent accessible surface area was calculated using FreeSASA (Mitternacht, 2016). The distance criterion is permissive, and residue pairs are retained only if they also bury solvent accessible surface area, which excludes residues that are not exposed in the isolated chain. The effect of these choices was assessed by varying the distance cutoff and the BSA threshold (Table S2).

For model training and validation, the dataset was randomly divided into training and validation subsets using an 80:20 split.

Primary model performance was evaluated using the test dataset introduced by Duarte et al. (2012). This benchmark contains carefully curated interfaces with experimentally validated classifications of biological assemblies and crystal contacts. After preprocessing, the dataset contained 162 interfaces in total, consisting of 81 biological interfaces and 81 crystal contacts. This dataset was used exclusively for testing and was not used during training or model selection. The datasets used in this study were obtained from the SBGrid data repository (https://data.sbgrid.org/dataset/843/), which hosts the benchmark datasets originally compiled by Renaud et al. (2021).

An additional evaluation was performed using the dataset introduced by Zhu et al. (Zhu et al., 2006). The Zhu dataset comprises 243 interfaces from 137 structures. Eight interfaces from four structures (1BOL, 1BYK, 1FGO, 1DNL) were excluded because the chain pair specified in the original dataset could not be unambiguously identified in the current PDB entry. The remaining 235 interfaces were retained for the sequence‐redundancy filtering described in the following section.

### 4.3 Control of sequence redundancy

Because structural datasets often contain homologous proteins, all models evaluated in this study were trained under a redundancy‐controlled training protocol. Sequence similarity between training and test chains was assessed at the chain level.

Training entries whose chains exceeded 30% sequence identity to any chain present in the test dataset were removed (Bernett et al., 2024; Hamp & Rost, 2015). This filtered out 6.3% of the training and validation datasets; a complete list of redundant entries is provided in Table S1 and their sequence identity distribution is shown in Figure S1. Models trained on this filtered dataset were used to assess performance in a setting where homologous relationships between training and testing structures were mitigated. Results obtained without redundancy control are provided in Table S6. The same chain‐level procedure was applied to the Zhu dataset, using the primary dataset prior to redundancy reduction as the reference set. Zhu interfaces containing a chain with greater than 30% identity to any chain in that set were excluded, leaving 126 (64 biological and 62 crystal contacts) of the original 235 interfaces for additional evaluation (Figure S2).

### 4.4 Structural representation of interfaces

Structural information was represented using surface‐based geometric features derived from molecular surfaces. Interfaces were represented as point clouds using the dMaSIF framework, a geometric deep learning approach (Sverrisson et al., 2020). Surface points were generated for each chain forming the interface, and local geometric descriptors were associated with each point to capture the shape and physicochemical properties of the protein surface. The resulting surface point clouds were processed by a hierarchical point‐cloud encoder based on PointNet++ (Qi et al., 2017). Similar to the original PointNet++ architecture, the encoder performs farthest‐point sampling and k‐nearest‐neighbor grouping across multiple stages in order to progressively aggregate local geometric information. The final structural representation of the interface was obtained by applying global pooling to the point features in the last layer.

### 4.5 Sequence representation using protein language models

Sequence‐derived features were obtained using the ESM‐C protein language model (Lin et al., 2023). Residue embeddings were extracted from the final layer of the pretrained ESM‐C 600M model. The language model parameters were kept frozen during training. For each interface, residues participating in the interaction were identified using the structural interface definition described above. Embeddings corresponding to these interface residues were extracted and aggregated using mean pooling. For interfaces consisting of two chains, pooled embeddings from each chain were concatenated to form the final embedded representation.

For comparison, the same feature‐extraction and interface‐localized pooling procedure was also applied using ESM‐2 (esm2_t33_650M) and Ankh‐base. In all cases, the pretrained language models remained frozen, allowing the effects of the underlying protein language model to be evaluated independently of the pooling and classification procedures.

Although these features originate from a sequence‐based language model, structural information remains necessary to identify the interface residues used for pooling. The resulting representation therefore reflects sequence properties localized to structurally defined interaction regions.

### 4.6 Multimodal model architecture

To integrate structural and sequence information, we constructed a multimodal neural network in which geometric surface features were combined with ESM‐C embeddings. Cross‐attention layers were used in the initial stage of the model to associate surface‐based features with residue‐level language model embeddings (Figure S3). The fused representation was subsequently processed by neural network layers to produce a single interface‐level prediction (Figure S3).

### 4.7 Logistic regression models

To assess the predictive power of sequence‐derived embeddings, we trained logistic regression models using pooled representations obtained from protein language models. As a sequence‐only baseline, we used the concatenated mean‐pooled embeddings of the full‐length sequences of the two interacting chains. In addition, we evaluated a structure‐based ESM model (ESpma), in which the input consisted of the concatenated mean‐pooled embeddings of interface residues from the two chains.

As a spatial control, we additionally constructed representations from non‐interface surface residues, defined as non‐interface residues with rASA ≥ 25% following (Yan et al., 2008). Relative solvent accessibility was computed with FreeSASA. For each chain, non‐interface surface residues were randomly sampled to match the number of interface residues, and their embeddings were aggregated using mean pooling. The pooled representations of the two chains were then concatenated and evaluated using the same classification procedure. Because the control residues were randomly sampled, sampling was repeated three times for each pLM. The test AUROC varied by less than 0.005 across draws. This control was used to determine whether the predictive signal was specifically enriched at the interaction interface rather than being generally present across the protein surface.

The models were implemented in scikit‐learn (Pedregosa et al., 2012), and hyperparameters were optimized with Optuna (Akiba et al., 2019) based on validation set performance. Among several conventional classifiers evaluated on the same pooled pLM embeddings, regularized logistic regression showed the most consistent validation performance and was therefore selected as the baseline model.

### 4.8 Model training

All neural network models were trained using binary cross‐entropy loss. The models were optimized using Adam with an initial learning rate of 1 × 10^−3^, weight decay of 1 × 10^−4^, and a batch size of 24. Training proceeded for a maximum of 50 epochs with early stopping applied when validation loss failed to improve for 10 consecutive epochs. The early stopping criterion was met in all runs before reaching the 50‐epoch maximum, suggesting that further training would not benefit model performance (Figures S4–S7).

### 4.9 Evaluation metrics and comparison methods

Model performance was evaluated using classification accuracy (ACC), Matthews correlation coefficient (MCC), sensitivity, specificity, and area under the receiver operating characteristic curve  (AUROC). Predictions were converted to binary labels using a fixed probability threshold of 0.5. ACC, MCC, sensitivity, and specificity were calculated as follows:

$$\mathrm{ACC}=\frac{\mathrm{TP}+\mathrm{TN}}{\mathrm{TP}+\mathrm{TN}+\mathrm{FP}+\mathrm{FN}},$$

$$\mathrm{MCC}=\frac{\mathrm{TP}\;\mathrm{TN}-\mathrm{FP}\;\mathrm{FN}}{\sqrt{\left(\mathrm{TP}+\mathrm{FP}\right)\left(\mathrm{TP}+\mathrm{FN}\right)\left(\mathrm{TN}+\mathrm{FP}\right)\left(\mathrm{TN}+\mathrm{FN}\right)}},$$

$$\text{Sensitivity}=\frac{\mathrm{TP}}{\mathrm{TP}+\mathrm{FN}},$$

$$\text{Specificity}=\frac{\mathrm{TN}}{\mathrm{TN}+\mathrm{FP}},$$

where *TP*, *TN*, *FP*, and *FN* denote true positives, true negatives, false positives, and false negatives, respectively. Differences in AUROC between models were assessed using DeLong's test for two correlated ROC curves, with each model compared against the corresponding ESpma model (DeLong et al., 1988; Robin et al., 2011).

Our models were compared with previously reported methods for distinguishing biological assemblies from crystal contacts, including PISA (Krissinel & Henrick, 2007), EPPIC (Duarte et al., 2012), PRODIGY‐crystal (Jiménez‐García et al., 2019), DeepRank (Renaud et al., 2021), DeepRank‐GNN (Réau et al., 2023), and DeepRank‐GNN‐esm (Xu & Bonvin, 2024). PISA, EPPIC, and PRODIGY‐crystal were evaluated directly using available servers (accessed March 2026). EPPIC predictions were obtained using sequence information from UniProt (UniProt Consortium, 2025) release 2026_01. Because this method was developed using the primary test dataset, the present evaluation should be regarded as a consistency test rather than an independent external benchmark. The DeepRank family and PRODIGY‐crystal were developed using the same training set as the models in this study, although without the redundancy control applied here. DeepRank‐GNN‐esm was run locally using the published weights. PISA returns a discrete assignment by design. For the original DeepRank, continuous prediction scores are not reported and trained weights for this are not publicly available, so predictions could not be regenerated. These methods were therefore included as reference points based on their binary predictions or reported confusion‐matrix‐based metrics (Renaud et al., 2021). For DeepRank‐GNN, published prediction scores were used to calculate performance metrics using the same evaluation procedure as for our models.

### 4.10 Per‐residue contribution

Because mean pooling and the logistic‐regression decision function are both linear, the logit decomposes exactly into a sum of per‐residue terms. For residue *i*, the relative contribution is defined as *cᵢ* = *eᵢ* · *w*, where *eᵢ* is its ESM‐C embedding and *w* is the corresponding half of the weight vector.

## DISCLOSURE

The authors used GitHub Copilot, ChatGPT (OpenAI), and Claude (Anthropic) as programming aids during software development and code revision. All generated code was critically reviewed, tested, and modified by the authors, who take full responsibility for the final implementation and all scientific conclusions.

## AUTHOR CONTRIBUTIONS

**Yoshinori Fukasawa:** Conceptualization; writing – review and editing; supervision; investigation; resources. **Sarah Nozawa:** Writing – original draft; methodology; software; formal analysis; investigation; visualization. **Kentaro Tomii:** Funding acquisition; writing – review and editing; project administration; investigation.

## Supporting information


**Figure S1.** Distribution of maximum sequence identity between training (including validation) and chains in the primary test dataset.
**Figure S2.** Distribution of maximum sequence identity between Zhu chains and chains in the training (including validation dataset) dataset before the redundancy removal.
**Figure S3.** Architecture of the multimodal ESpma_pc_ model. Surface geometry of the interface is encoded from point clouds computed by dMaSIF using a PointNet++‐based encoder, while interface residues are represented using ESM‐C embeddings. The structural and sequence representations are fused to classify an interface.
**Figure S4.** Training and validation loss during model training for the PC model. The training and validation loss on the original dataset are shown as a function of training epochs.
**Figure S5.** Training and validation loss during model training for the PC model. The training and validation loss on the sequence redundancy mitigated dataset are shown as a function of training epochs.
**Figure S6.** Training and validation loss during model training for the ESpma_pc_ model. The training and validation loss on the original dataset are shown as a function of training epochs.
**Figure S7.** Training and validation loss during model training for the ESpma_pc_ model. The training and validation loss on the sequence redundancy mitigated dataset are shown as a function of training epochs.

**Table S1.** Complete list of redundant entries.
**Table S2.** Sensitivity of ESpma performance to alternative interface‐residue definitions.
**Table S3.** Performance of methods for which only confusion‐matrix‐based evaluation was feasible.
**Table S4.** Performance of pooling strategies on the Zhu dataset across three pLM backbones.
**Table S5.** Predictive performance of SAE‐reconstructed and residual representations.
**Table S6.** Performance of pooling strategies when trained without redundancy control.

## Data Availability

The code used in this study is publicly available at https://github.com/fukasawa-group/espma. A Google Colab link for running the method is also available in the repository.
