# Supplementary material for: ESpma: A method for assessing biological/non‐biological interfaces using point‐clouds‐based structural features and protein language models
Source: Protein Sci. 2026 Sep 17;35(10):e70790. doi: 10.1002/pro.70790 (PMC13584886; doi:10.1002/pro.70790)
Supplement: Supplementary file 1 [file PRO-35-e70790-s002.pdf]

Supplementary Material

ESpma: a Method for assessing Biological/non-Biological Interfaces using Point-clouds-based Structural Features and Protein Language Models

Sarah Nozawa[1], Kentaro Tomii[2], Yoshinori Fukasawa[1]

[1]Center for Bioscience Research and Education, Utsunomiya University, Utsunomiya, Japan

[2]Artificial Intelligence Research Center, National Institute of Advanced Industrial Science and Technology (AIST), Tokyo, Japan

Tables S1-S6 (TablesS1-S6.xlsx) and Figures S1-S7.
Table S1 lists entries removed by redundancy control. Table S2 reports performance under alternative interface definitions. Table S3 reports methods without continuous prediction scores. Table S4 reports performance on the Zhu dataset across three pLM backbones. Table S5 reports performance of the sparse autoencoder models. Table S6 reports performance when trained without redundancy control. Figures S1 and S2 show the distribution of sequence identity for the training and Zhu datasets, respectively. Figure S3 illustrates the detailed architecture of the multimodal $ESpma_{pc}$. Figures S4-S7 show the training and validation loss curves.

Supplementary Table S1. Complete list of redundant entries (see TablesS1-S6.xlsx)**.**

List of the redundant entries removed in the training set whose chains exceeded 30% sequence identity to any chain present in the primary test dataset. For the Zhu dataset, entries were compared against the training dataset prior to redundancy reduction.

Supplementary Table S2. Sensitivity of ESpma performance to alternative interface-residue definitions.

Interface residues were defined using combinations of heavy-atom distance cutoffs (5 or 8 Å) and buried surface area (BSA) cutoffs (≥1 or ≥10 Å²). Conditions without a BSA cutoff were also evaluated. Pairwise differences in AUROC relative to the original interface definition (8 Å and BSA ≥1 Å²) were assessed using DeLong's test.

BSA cutoff	Distance cutoff	ACC	AUROC	*p*-value
≥10 Å²	5 Å	0.864	0.912	0.511
≥10 Å²	8 Å	0.864	0.91	0.810
≥1 Å²	5 Å	0.858	0.913	0.105
≥1 Å²	8 Å	0.858	0.909	-
none	5 Å	0.84	0.91	0.939
none	8 Å	0.79	0.89	0.049

ACC, accuracy; AUROC, area under the receiver operating characteristic curve; Biological interfaces were treated as the positive class. *P*-values were calculated using DeLong's test by comparing each AUROC with the interface definition applied in this study (8 Å and BSA ≥1 Å²).

Supplementary Table S3. Performance of methods for which only confusion-matrix-based evaluation was feasible.

Continuous prediction scores were unavailable for PISA, and reproducible continuous prediction scores were unavailable for the published implementation of DeepRank. Consequently, only confusion-matrix-derived performance metrics are reported.

	Sen	Spe	ACC	MCC
PISA	0.963	0.568	0.765	0.578
DeepRank	0.880	0.837	0.857	0.716

Sen, sensitivity; Spe, Specificity; ACC, accuracy; MCC, Matthews correlation coefficient; AUROC, area under the receiver operating characteristic curve; Biological interfaces were treated as the positive class.

Supplementary Table S4. Performance of pooling strategies on the Zhu dataset across three pLM backbones.

Classification performance on the Zhu dataset (64 biological and 62 crystal) for full-sequence pooling, non-interface surface pooling, and interface-localized pooling (ESpma), using ESM-C 600M, ESM-2 650M, and Ankh-base embeddings. All models were trained on the primary redundancy-controlled benchmark and evaluated here using an identical procedure. Results for the ESM-C backbone are reproduced in Table 4.

Method	Sen	Spe	ACC	MCC	AUROC	*p*-value
ESM-C						
Baseline1	0.359	0.565	0.460	-0.078	0.428	<0.001
Baseline2	0.516	0.613	0.563	0.129	0.615	<0.001
ESpma	0.672	0.935	0.802	0.628	0.938	-
ESM-2						
Baseline1	0.266	0.742	0.500	0.009	0.473	<0.001
Baseline2	0.422	0.758	0.587	0.191	0.627	<0.001
ESpma	0.563	0.984	0.770	0.600	0.919	-
Ankh						
Baseline1	0.281	0.661	0.468	-0.062	0.463	<0.001
Baseline2	0.422	0.661	0.540	0.086	0.558	<0.001
ESpma	0.594	0.952	0.770	0.582	0.921	-

Abbreviations are as defined in Table S2. Baseline1: Mean pooling over the full-length sequences of the two interacting chains. Baseline2: Mean pooling over randomly sampled non-interface surface residues, matched to the number of interface residues. ESpma: Mean pooling over structurally defined interface residues. *P*-values were calculated using DeLong's test by comparing each AUROC with that of the corresponding ESpma model.

Supplementary Table S5. Predictive performance of SAE-reconstructed and residual representations.

Classification performance obtained by pooling SAE-reconstructed ESM-C embeddings and the residual embeddings defined as the difference between the original and reconstructed representations.

Method	Sen	Spe	ACC	MCC	AUROC	*p*-value
ESpma (original)	0.778	0.914	0.846	0.698	0.912	-
ESpma-SAE-reconstructed	0.605	0.926	0.765	0.561	0.838	0.011
ESpma-SAE-residual	0.605	0.926	0.765	0.561	0.842	0.016

Abbreviations are as defined in Table S2. *P*-values were calculated using DeLong's test by comparing AUROC with that of ESpma (original) model.

Supplementary Table S6. Performance of pooling strategies when trained without redundancy control.

Classification performance on the primary benchmark for the three pooling strategies across three pLM backbones, with models trained on the full training and validation sets. The test set was identical in both settings. Redundancy-controlled results are given in Table 1.

Method	Sen	Spe	ACC	MCC	AUROC	*p*-value
ESM-C						
Baseline1	0.568	0.963	0.765	0.578	0.834	0.012
Baseline2	0.617	0.864	0.741	0.497	0.816	0.002
ESpma	0.802	0.914	0.858	0.721	0.909	-
ESpmapc	0.852	0.840	0.846	0.691	0.915	0.187
ESM-2						
Baseline1	0.593	0.963	0.778	0.598	0.846	0.047
Baseline2	0.654	0.914	0.784	0.588	0.861	0.157
ESpma	0.778	0.926	0.852	0.712	0.898	-
Ankh						
Baseline1	0.457	0.938	0.698	0.451	0.819	0.017
Baseline2	0.543	0.877	0.710	0.445	0.809	0.018
ESpma	0.778	0.914	0.846	0.698	0.892	-

Abbreviations are as defined in Table S2. P-values were calculated using DeLong’s test by comparing AUROC with that of ESpma within each pLM backbone.

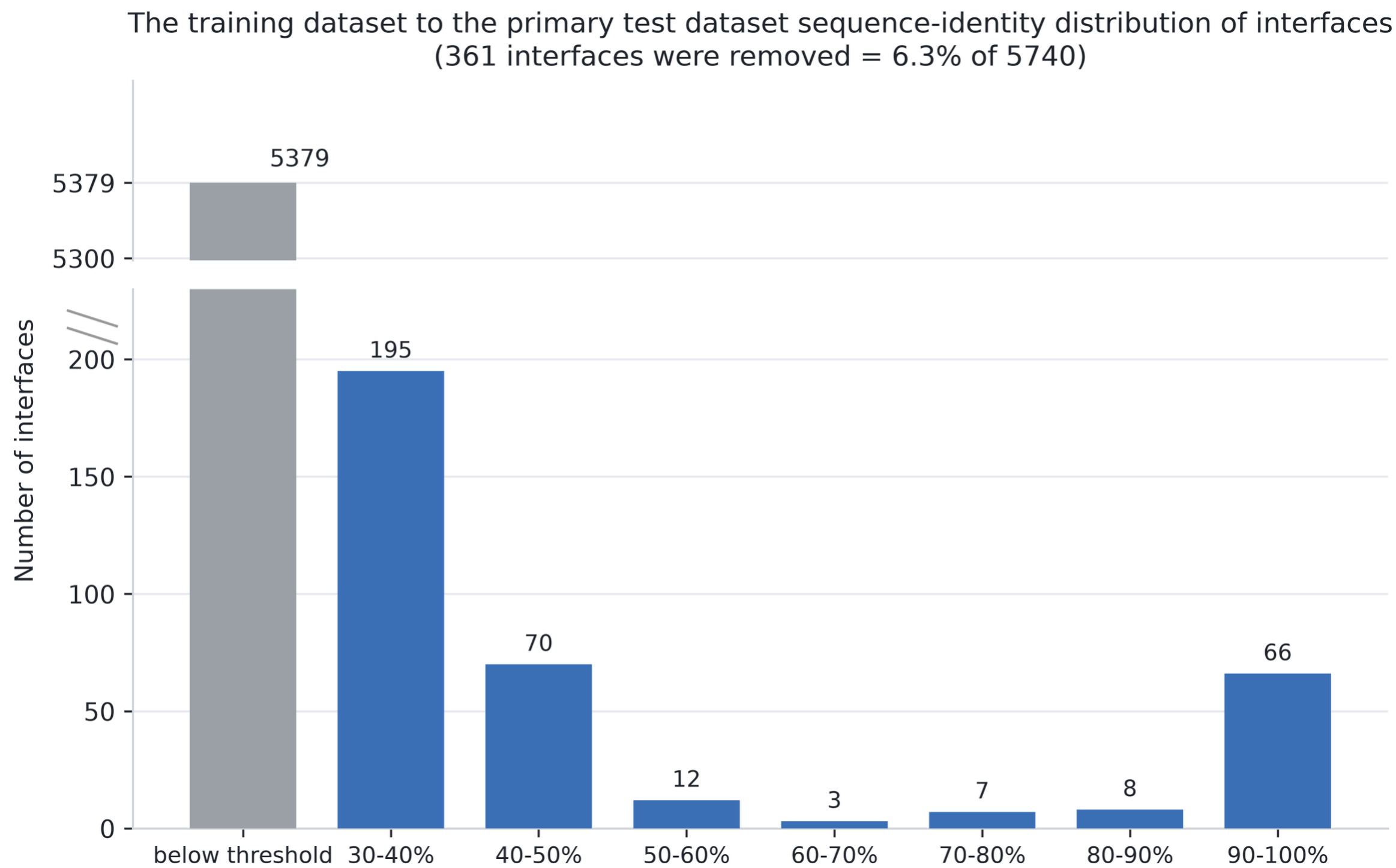

Supplementary Figure S1. Distribution of maximum sequence identity between training (including validation) and chains in the primary test dataset.

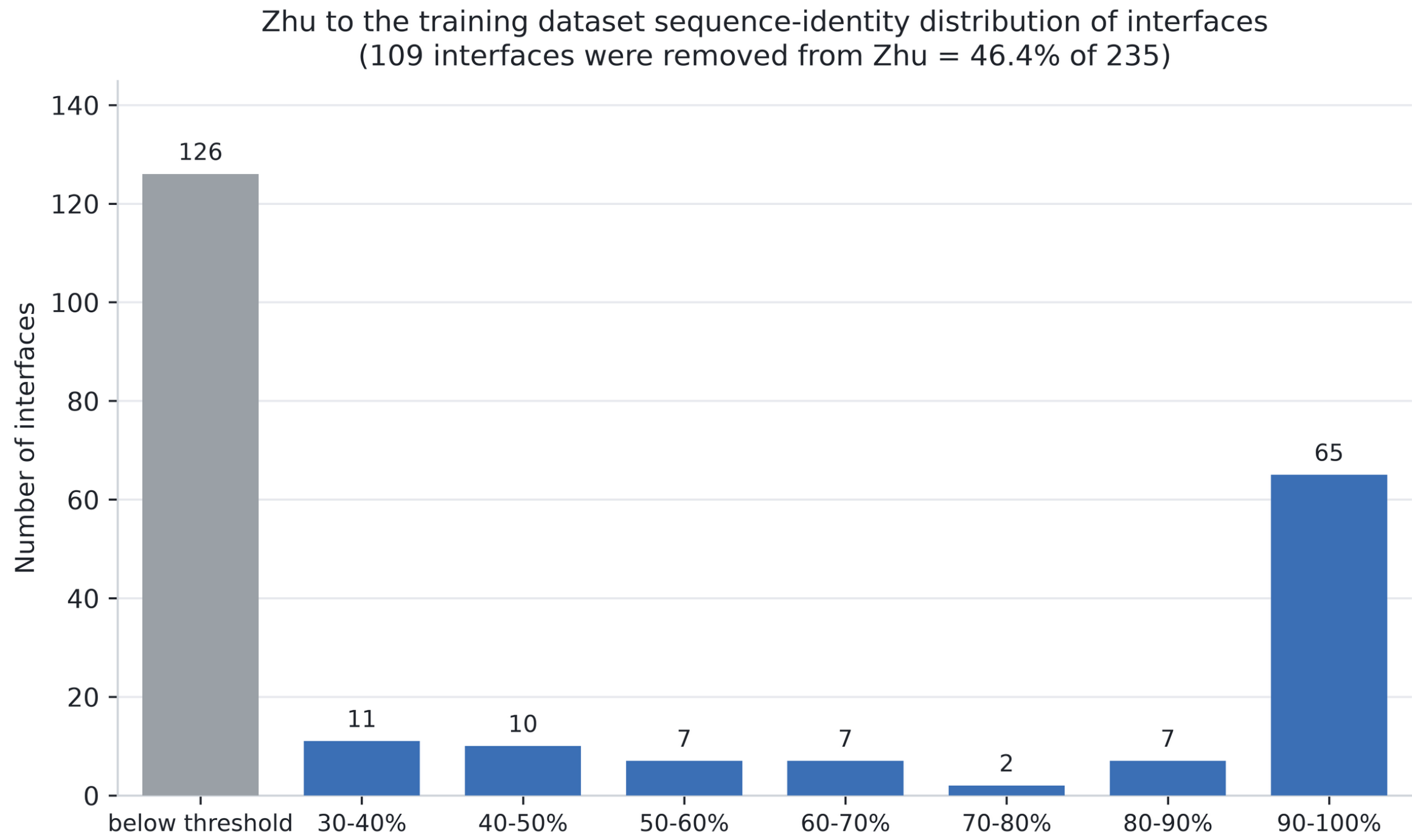

Supplementary Figure S2. Distribution of maximum sequence identity between Zhu chains and chains in the training (including validation dataset) dataset before the redundancy removal.

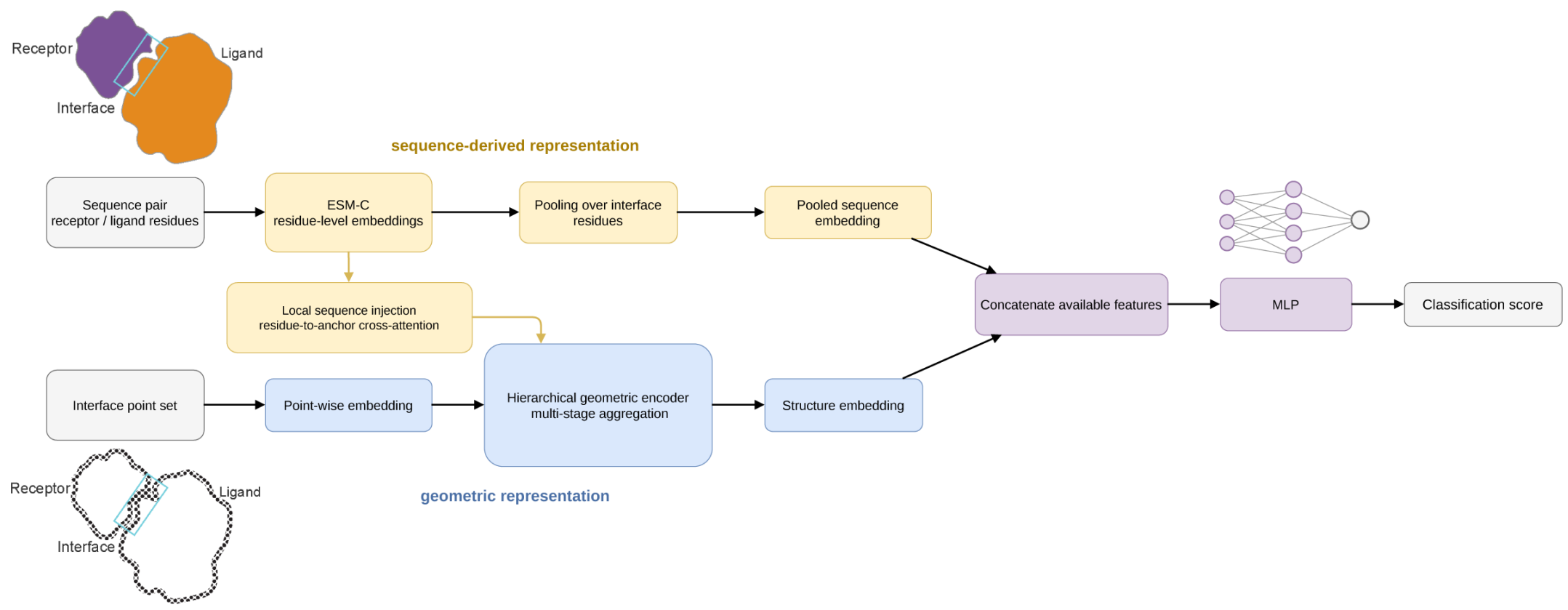

Supplementary Figure S3. Architecture of the multimodal ESpma$_{pc}$ model. Surface geometry of the interface is encoded from point clouds computed by dMaSIF using a PointNet++-based encoder, while interface residues are represented using ESM-C embeddings. The structural and sequence representations are fused to classify an interface.

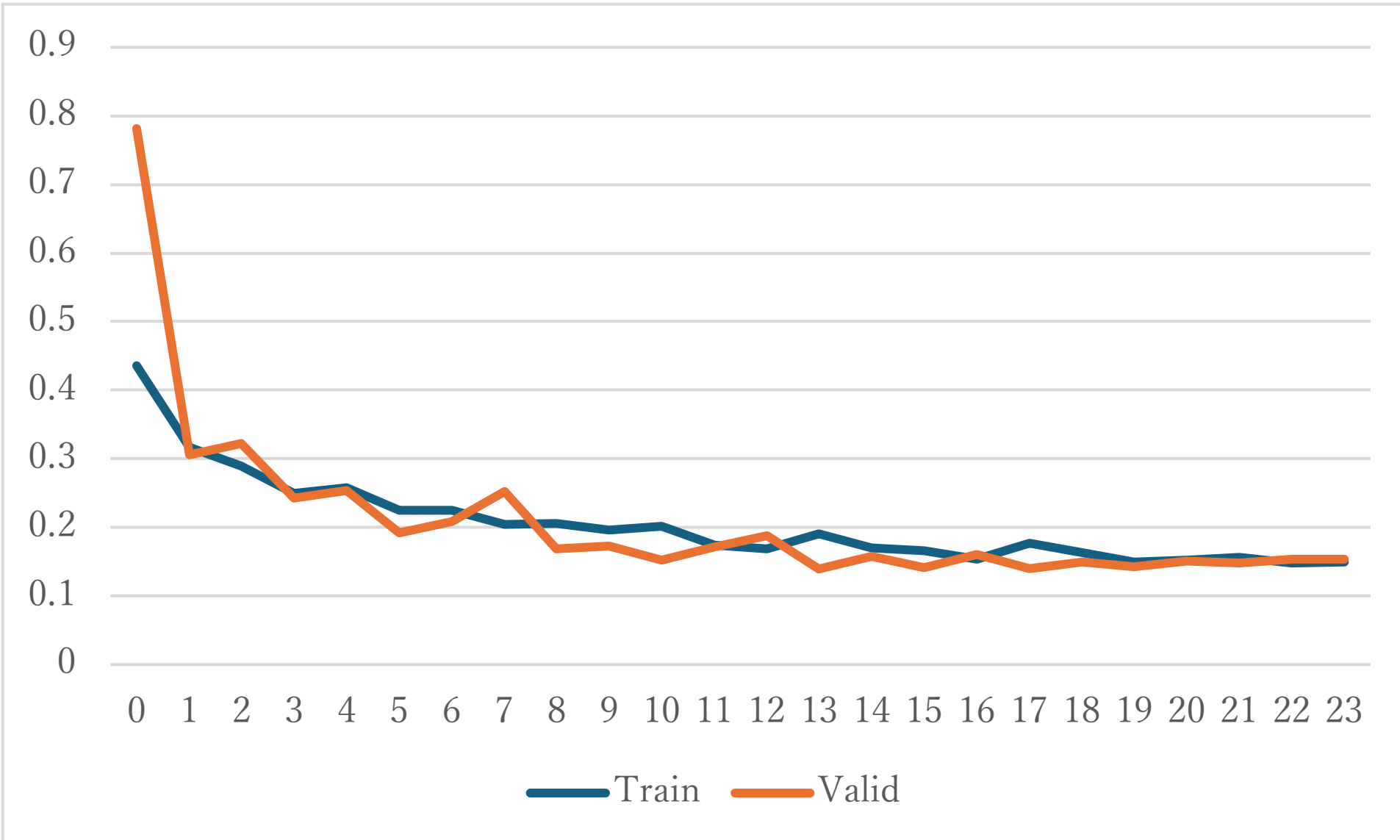

Supplementary Figure S4. Training and validation loss during model training for the PC model.

The training and validation loss on the original dataset are shown as a function of training epochs.

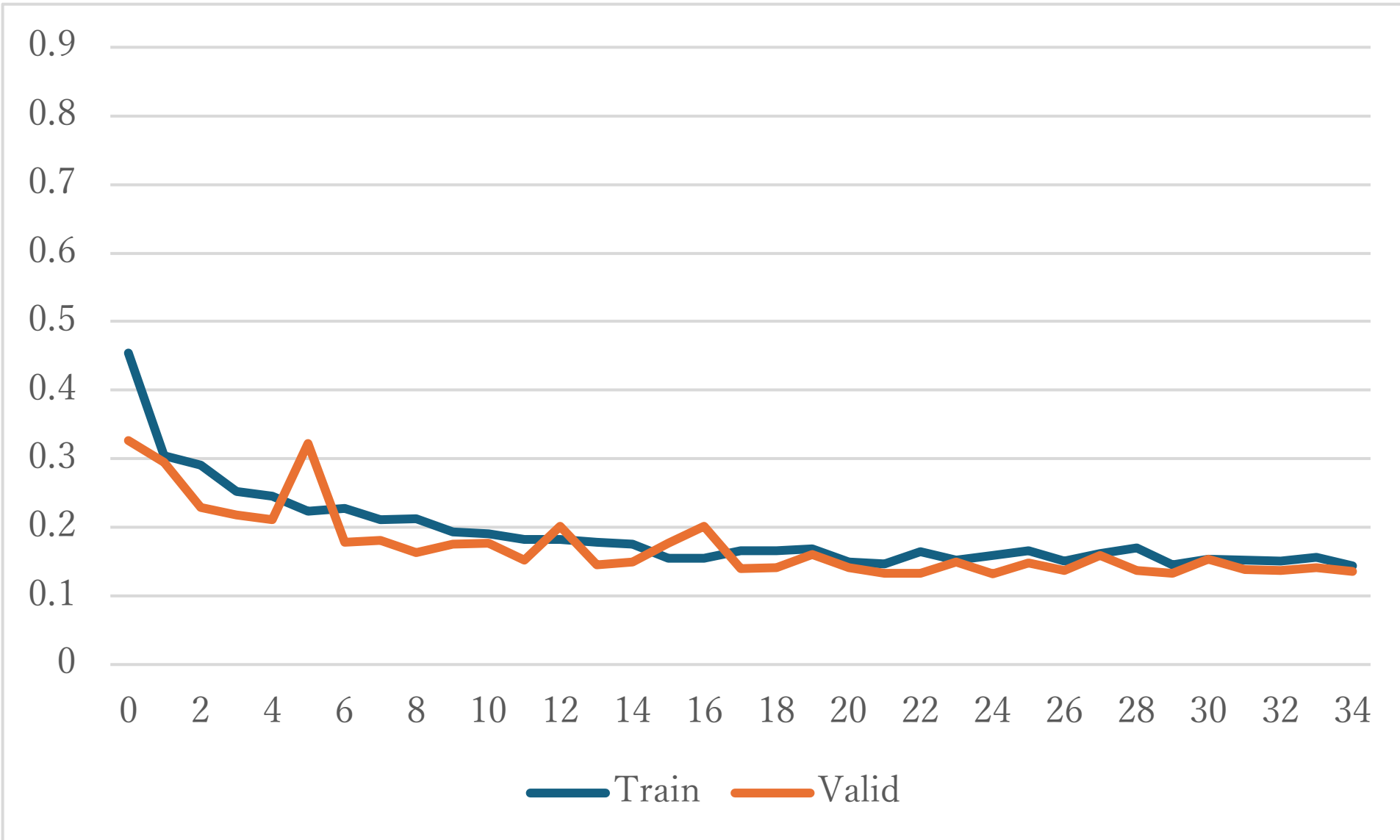

Supplementary Figure S5. Training and validation loss during model training for the PC model.

The training and validation loss on the sequence redundancy mitigated dataset are shown as a function of training epochs.

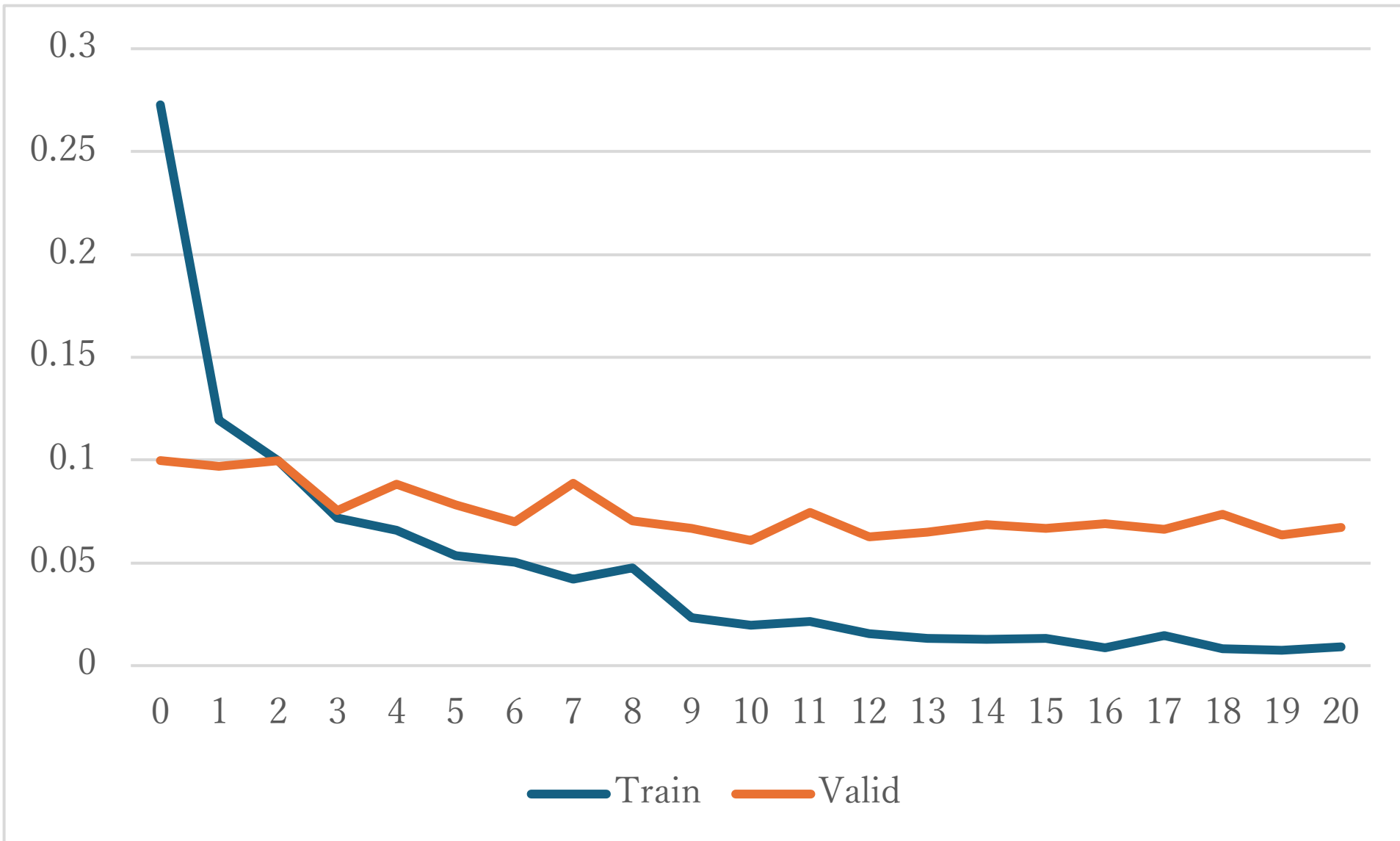

Supplementary Figure S6. Training and validation loss during model training for the ESpma$_{pc}$ model.

The training and validation loss on the original dataset are shown as a function of training epochs.

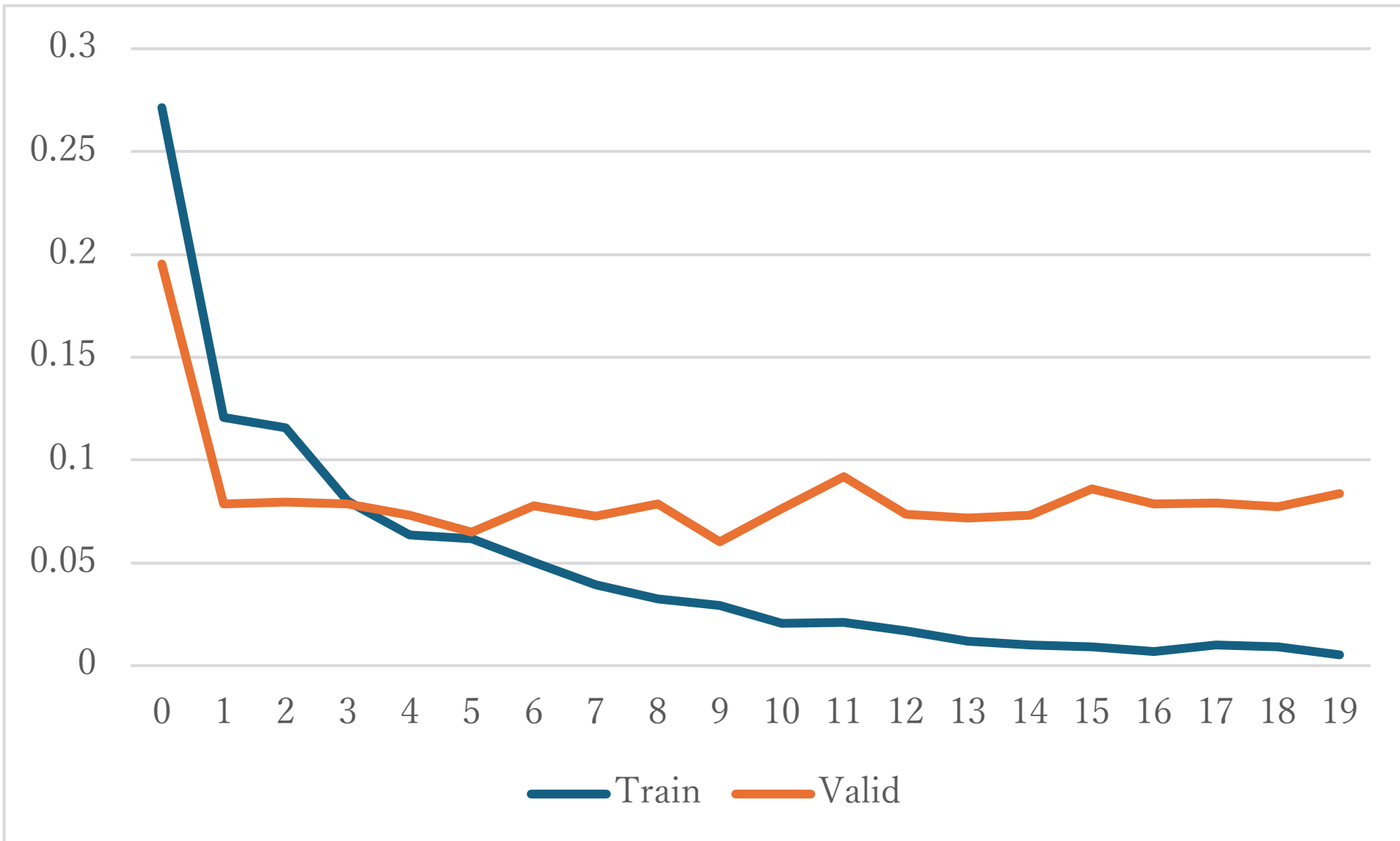

Supplementary Figure S7. Training and validation loss during model training for the ESpma$_{pc}$ model.

The training and validation loss on the sequence redundancy mitigated dataset are shown as a function of training epochs.
